# A CHANGING INDUSTRY IN A CHANGING WORLD: LONG-TERM SERVICES WHERE ARE YOU GOING?

**DOI:** 10.1093/geroni/igz038.2836

**Published:** 2019-11-08

**Authors:** Robert A Applebaum, Matt Nelson, Jane Straker, A Katherine Harrington, John R Bowblis

**Affiliations:** 1 Miami University, Scripps Gerontology Center, Oxford, Ohio, United States; 2 Scripps Gerontology Center at Miami University, Oxford, Ohio, United States; 3 Department of Economics, Miami University, Oxford, Ohio, United States

## Abstract

From its name to the type and setting of care provided, the world of long-term services and supports has changed dramatically in the last two decades. Using 26 years of longitudinal data from the state of Ohio this presentation describes how the long-term services system is different from the one that existed in the early 1990’s. Data come from 13 biennial surveys of Ohio nursing homes and residential care facilities (95% response rate) and comprehensive resident and home care participant data on user characteristics and utilization rates. Findings show large changes in where services are provided and who receives services. For example, Ohio, as has most other states, has changed the ratio of its older population using Medicaid long-term care, going from 91% nursing home users in 1993, to more than half using home and community-based services in 2017. At the same time the sheer number of admissions to Ohio nursing homes increased from 70,000 to more than 220,000 and Medic are admissions increasing from 30,000 to 145,000, painting a picture of today’s nursing home as a short-term care provider. These massive changes indicate an industry in transition. What will this mean for the future of the home care and nursing home industries? What will tomorrow’s system of long-term services and supports look like? Building on more than two decades of findings the presentation will tackle the question of where long-term services is going in the future.

